# General Surgery

**Published:** 1895-01-05

**Authors:** 


### GENERAL SURGERY.

(*Continued from page 223.*)

**Calculus of the Ureter.**—The diagnosis is made by a consideration of the symptoms described in the former article, aided, in thin subjects, by abdominal palpation. If the stone be impacted at the entrance of the ureter into the bladder it is almost always to be felt per rectum in the male, or per vaginam in the female aided by digital examination of the bladder. In two cases, those of Lane and Hall, the diagnosis has been confirmed by abdominal section.

He concludes as to treatment that it is immensely important to find out whether the stone has passed on into the bladder. Cases of unsuspected atrophy of kidney from impaction are only too frequent, and are too often only discovered by the occurrence of mischief on the other side leading to suppression. Where, therefore, there is a history of renal colic of intermittent character, combined with tenderness at one spot and no indication of the stone having passed on, operation is indicated.

In another class of cases where hydronephrosis or pyonephrosis exists, the stone may be removed before proceeding to more radical measures. When suppression of urine has already occurred the treatment may be considered under two heads—(1) Where uræmic poisoning is not well marked; (2) where pronounced. In the first case, thoroughly explore the ureter and remove the stone. If none be found, cut down on the kidney and put in a drainage tube. In the second event it is no good searching for the stone, as the patient's condition will not bear it, but open the kidney and drain off the urine. If the uræmia is relieved a search for the stone may be made afterwards.

If the calculus be impacted at the upper end of the ureter, the symptoms resemble calculus in the kidney, and nephrotomy is sure to be performed. The stone may then be found by enlarging the wound if necessary and passing a soft bougie down the tube to locate the stone. He thinks Godlee's idea of pushing the stone down the ureter into the bladder by means of an œsophageal bougie dangerous, as ulceration may have occurred. He points out that the middle three-fifths of the ureter may be explored by an operation similar to that for ligature of the common iliac artery. The peritoneum is stripped up and carries the ureter forward with it, then the tube may be explored with finger and thumb. If found low down, the stone may be worked up towards the wound with finger and thumb and extracted. The removal of a calculus at the lower end is easy through the roof of the vagina in the female. He does not agree with Morris as to the advisability of enlarging the ureteral opening at the lower end and extracting by forceps, as there is a danger of opening the peritoneum. Also more than one stone is frequently found in this situation. The only death recorded occurred where this operation was practised. Although no case of operation is yet recorded in the male for impaction at the lower end, yet cases have been observed. He advocates a perinæal operation, dissecting down between urethra and rectum to base of bladder, which he thinks would be preferable to the severe operation of Kraske, in which the coccyx and part of the sacrum are removed. He finds there is no need to suture the ureter after incising it, owing to its thickness and expansile properties; indeed, it constitutes a source of danger to do so.

A case has been recorded by Penrose[9] in which, in performing abdominal hysterectomy for carcinoma uteri, with an extensive infiltration into the left broad ligament, he was obliged to excise an inch of the left ureter to remove all diseased tissue. After removing the uterus, and suturing the peritoneum over the vaginal opening, he ligated the distal end of the ureter and implanted the proximal end in the bladder after Van Hook's method. Abdomen was closed without drainage, and the patient made an uninterrupted recovery, passing thirty-eight ounces of urine on the fourth day, and being discharged on the twentieth day.

**Surgery of Bladder.**—Weir[10] has placed on record cases which will show the marked advances made of late in genito-urinary surgery. As regards the bladder, he remarks that of its tumours quite 80 per cent. are malignant. They are but seldom situated at the summit or on the anterior face of the bladder or in the trigone, but the region between the summit and the mouths of the ureter, and especially on the posterior wall is the usual site. One-half of the cases of carcinoma, however, invade one or both of the ureteral orifices. Secondary growths occur quite late in the disease, though adjacent gland infection is not uncommon; but the latter is usually situated within surgical reach. The mortality involved by the removal of a carcinomatous growth of the bladder reaches, according to Alburan, to nearly 50 per cent. Till quite recently the usual method for removing vesical tumours has been to expose the growth by suprapubic section of the bladder, with its subsequent removal by knife or scissors, the interference seldom going beyond the mucous or muscular layers at the base of the growth. Recurrence after this method amounts to 57 per cent. Sonnenburg in 1884, first adopted a more heroic method, excising nearly the entire bladder except the neck, trigone, and ureteral orifices. The patient lived five weeks after the operation, and at the autopsy a new but comparatively small bladder, formed of the remnant of that organ and a modified cicatricial tissue was found. In 1885 Antal made a further step in advance. It consisted in the demonstration that the peritoneum could be peeled off from the summit and posterior wall of the bladder without much difficulty. Since that time nearly 20 cases of resection of more or less considerable portions of the bladder walls outside the limit of malignant growths have been undertaken. The benefit that accrues from this wider removal of a malignant growth is seen at once in the diminished chances of recurrence after such operations. From 57 per cent. it has been reduced to 28 per cent. With the encouragement afforded by this improvement in the technique of the operation larger and larger portions have been removed, and even entire ablation has been successfully undertaken. Bardenheuer in 1887 did this for cancer of the base involving the ureters. This was readily accomplished, but death resulted from uræmia. Two other extensive resections, though not total ones, have since been reported by the same surgeon, one of which has remained completely cured up to the date of the last report, and one died five months later from a recurrence. The difficulty connected with such extreme operations, apart from the immediate results of shock, was centred in the disposition of the severed ureters. Bardenheuer, in his experiments on dogs, found that implantation into the rectum was followed by death, due either to dilatation of the ureters following a contraction at the place of implantation, or in consequence of an infectious pyonephrosis of rectal origin. Similar fatal results have occurred in the human subject in nearly every instance where this implantation into the rectum has been resorted to. The vagina has been used in women as a retentive sac for the urine with some satisfaction. Le Dentu and Pozzi in the male brought the kidney end of the ureter to the abdominal wall. The former's patient died on the thirteenth day, when no ureteral or kidney changes were found. In Pozzi's case recovery took place, and urination through the abdominal wall was endured for three months, when the discomfort of it demanded a nephrectomy. In the future probably such expedients will be less resorted to, since by Van Hook's and Kuster's methods better results may be looked forward to. But a better method exists. De Paoli and Brusachi have shown that if a small portion of the bladder be left the ureters can be inserted into that portion, without subsequent contraction, by first making an opening through the thickness of the bladder, cutting away around this opening a small circle of the mucous membrane, and then pushing through the opening in the bladder wall the end of the ureter. The orifice of this is enlarged by splitting it up a short distance, as is usually done in the urethra in amputation of the penis, and then stitching the ureteral mucous membrane to the mucous membrane of the bladder. External to the bladder wall this fixation is reinforced by other sutures. This has been successfully resorted to in the human subject by Penrose, of Philadelphia. Kummel, still bolder, after the entire removal of the bladder attached the ureteral orifice to the vesical end, but his patient did not live long enough to test the procedure.

Weir uses a rectal bag, filled with 4 to 6 oz. and a variable amount of Thiersch's solution in the bladder, *i.e.*, until the summit is well raised above the pubes, a three-inch incision in the median line, and the bladder is opened by a vertical incision.

Vigneron states[11] that a solution of antipyrine, injected into the bladder, exercises an analgesic action sufficiently powerful to suppress or mitigate the painful sensations and contractions resulting from medicinal irrigations and instillations of nitrate of silver. He considers that it is indicated in all cases of cystitis in which the topical treatment gives rise to pain. He advises injecting into the undistended bladder, before resorting to irrigation, 2½ to 5 drachms of a 4 per cent. solution, ten minutes being allowed to elapse to get the full benefit of the remedy. If the bladder is distended, as in many prostatic cases, he leaves in 2 to 4 oz. of ½ or 1 per cent. solution after irrigation.

Keegan[12] gives a further series of 64 cases of litholapaxy performed on male children and boys during a period of seventeen months. In all, this surgeon has performed 239 litholapaxies, with but five deaths. Out of 68 cases he was obliged to cut in four, three of these being lateral lithotomies, and the remaining case a suprapubic cystotomy.

Yvon[13] suggests a new method of treating calculi by electrolysis. He finds that when a galvanic current is passed by a platinum electrode into an aqueous solution of sulphate of sodium, sulphuric acid and oxygen are disengaged at the positive pole, and at the negative pole sodium, which gives caustic soda and hydrogen. If a urinary calculus is placed between the points of the two platinum electrodes, isolated from each other and arranged in the form of a pair of forceps, and the whole is then plunged into an aqueous solution of sulphate of sodium, it is found that, under the influence of the electric current, the calculus is dissolved and pierced at the point of contact with the electrodes. The solution is effected at the positive pole if the calculus be composed of triple phosphates or earthy carbonates; at the negative pole if of uric acid.

He thinks that this might be carried out in the human subject. The bladder having been distended with the sulphate of sodium solution, the calculus might be seized with a pair of forceps arranged like a lithotrite, the two blades holding the platinum points isolated from each other. Solution of the calculus would then be gradually effected.

Lauenstein[14] has recently contributed to our knowledge of the rare accident of strangulation of the testicle by torsion of the spermatic cord. The fact that the cases recorded varied between the ages of four and sixty-two years of age, would indicate that age has little to do with this disease. All of these testicles must have been abnormally moveable. In five cases there was a distinct exciting cause. A jump over a ditch on the previous day, a fall of 12 feet three days before, sneezing, and hard manual labour were the causes assigned. In all the attack came on suddenly, and the pain was invariably severe. The torsion of the cord presented the same picture in all cases. The testicle was shiny, darkly discoloured, more or less swollen, and surrounded by yellow or bloody fluid. The testicle spontaneously untwisted as soon as it was relieved from constricting influence. The hæmorrhagic infarction was in all cases unquestionably due to twisting of the pedicle. Kocher thinks that the separation of the funiculus into two segments has much to do with causing this accident. In the Mikulicz case it is stated that the tail of the epididymis had become twisted about the first portion of the vas deferens. Lauenstein thinks that shortness of the cord may have something to do with the accident. In Nicoladoni's first case the cord, which was twisted 180 degrees, measured but two centimetres long. The flat form of the testicle may also have something to do with the occurrence, or rather with the maintenance of this deformity. In five out of six cases, where the point was noticed, there was a left spiral twist. Four of the cases involved undescended testes of the right groin, and one a testicle just outside the outer ring.

Of chief importance is the diagnosis. In all cases the symptoms simulated some inflammatory process. The onset was very sudden, and followed by pain, local swelling, œdema, redness, and fever. The sudden onset was usually followed by reflex disturbance, especially vomiting. The diagnosis of torsion of the testicle, on account of the suddenness of the attack and the possible co-existence of hernia of the intestine or omentum, may be confused with that of the latter condition. Abscess of the appendix vermiformis is also to be borne in mind.

A case of abscess of the seminal vesicles has been treated by Von Dittel[15] in the following manner: A young man, twenty years of age, was the subject of blennorhagia at the beginning of January, 1894, and on the the 20th orchitis developed. The temperature was 101·3 deg. Fahr. in the morning, and 104 deg. in the evening. By the rectum, the region behind the prostate, at the level of the seminal vesicles, was soft to the touch and sensitive to pressure, which, when persisted in, caused a discharge of pus from the urethra. The patient complained little of the region attacked, but often of a sensation of pressure, sometimes with sharp radiating pains, painful defæcation, and painful ejaculation of reddish-brown semen, pus, and blood. Van Dittel opened the abscess by making a perinæal incision. The rectum was bent down to the left, the coccyx cut off, and the suppurating part cleared out and immediately washed. After several days of fever the patient recovered, and at the end of a month he was cured. This method has the advantage that it can be carried out antiseptically, which cannot be done with simply puncturing or incising the rectum and draining it.

Manley[16] has contributed a thoughtful article on gonorrhœal arthritis, and makes some valuable remarks on diagnosis. If one is not cautious the disease may be confounded with rheumatism or tubercular disease. It is unlike rheumatism in many cardinal features. First, there is not the same intense, fluctuating pyrexia or exhaustive perspiration; the urine is free from the excessive deposits and saturation with phosphates. Cardiac involvement is unusual. The disease is generally monarticular; its onset is gradual. It is said to be most frequent in the male (Manley says it is more frequent in women than is generally believed) and most frequently attacks the knee-joints. The one symptom more common and valuable than all others is the persistence of pain and inflammation in one articulation. Another is the chronicity of the effusion into the joints. Unlike rheumatism, surface hyperæsthesia is not as a rule present, so that in order to elicit pain some slight pressure is needed. In gonorrhœal arthritis the joint is immovably fixed and cannot be moved for a considerable period without great pain. The soft parts above and below the articulation are very sensitive. To the sense of touch the surface temperature is much less than in rheumatism. Rheumatic inflammation never runs into suppurative changes within the articulation. But in severe forms of gonorrhœal arthritis suppurative inflammation is not uncommon. In gonorrhœal arthritis colchicum and the alkalies make no impression. White, of Philadelphia, recommends energetic medication at the outset. He reports excellent results by the free use of quinine and the biniodide of mercury, the former in doses of five to ten grains three times a day, along with one-tenth grain doses of the latter, until an impression is made on the disease. Locally, the early application of ten to twelve leeches to the joint is most useful.

[9] Kansas City Med. Index, 1894, No. 5. [10] New York Med. Rec., August 11, 1894. [11] Med. Week, June 8, 1894. [12] Lancet, July 28, 1894. [13] Med. Week, June 22, 1894. [14] Annals of Surgery, July, 1894. [15] New York Med. Jour. July 14, 1894. [16] American Jour. of Med., July, 1894.

